# Role of monocytes and dendritic cells in cardiac reverse remodelling after cardiac resynchronization therapy

**DOI:** 10.1186/s12872-023-03574-4

**Published:** 2023-11-15

**Authors:** Sílvia Martins, Natália António, Ricardo Rodrigues, Tiago Carvalheiro, Cândida Tomaz, Lino Gonçalves, Artur Paiva

**Affiliations:** 1 Health Sciences Research Centre, University of Beira Interior (CICS-UBI), 6200-506 Covilhã, Portugal; 2 Instituto Politécnico de Castelo Branco, ESALD-Dr. Lopes Dias Health School, Ciências Biomédicas Laboratoriais, Castelo Branco, Portugal; 3 Department of Clinical Pathology, Centro Hospitalar Universitário Cova da Beira, Quinta Do Alvito, 6200-251 Covilhã, Portugal; 4 University of Coimbra, Coimbra Institute for Clinical and Biomedical Research (iCBR), Faculty of Medicine, Coimbra, Portugal; 5 Cardiology Department, Centro Hospitalar E Universitário de Coimbra, Coimbra, Portugal; 6 Institute of Pharmacology and Experimental Therapeutics/iCBR, Faculty of Medicine, University of Coimbra, Coimbra, Portugal; 7 Centro Do Sangue E da Transplantação de Coimbra, Instituto Português Do Sangue E da Transplantação, Coimbra, Portugal; 8 Chemistry Department, University of Beira Interior, Covilhã, Portugal; 9 Department of Clinical Pathology, Flow Cytometry Unit, Centro Hospitalar E Universitário de Coimbra, Coimbra, Portugal; 10 Instituto Politécnico de Coimbra, ESTESC-Coimbra Health School, Ciências Biomédicas Laboratoriais, Coimbra, Portugal; 11 Unidade Funcional de Citometria de Fluxo, Centro Hospitalar E Universitário de Coimbra, Praceta Mota Pinto, 3000-075 Coimbra, Portugal

**Keywords:** Chronic heart failure, Cardiac resynchronization therapy, Immune response, Monocytes, Dendritic cells, CD86

## Abstract

**Background and aims:**

Monocytes and dendritic cells (DC) are both key inflammatory cells, with recognized effects on cardiac repair. However, there are distinct subsets of monocytes with potential for beneficial or detrimental effects on heart failure (HF) pathogenesis. The connection between reverse cardiac remodelling, the potential anti-inflammatory effect of cardiac resynchronization therapy (CRT) and monocytes and DC homeostasis in HF is far from being understood. We hypothesized that monocytes and DC play an important role in cardiac reverse remodelling and CRT response. Therefore, we aimed to assess the potential role of baseline peripheral levels of blood monocytes and DC subsets and their phenotypic and functional activity for CRT response, in HF patients. As a secondary objective, we aimed to evaluate the impact of CRT on peripheral blood monocytes and DC subsets, by comparing baseline and post CRT circulating levels and phenotypic and functional activity.

**Methods:**

Forty-one patients with advanced HF scheduled for CRT were included in this study. The quantification and phenotypic determination of classical (cMo), intermediate (iMo) and non-classical monocytes (ncMo), as well as of myeloid (mDC) and plasmacytoid DC (pDC) were performed by flow cytometry in a FACSCanto™II (BD) flow cytometer. The functional characterization of total monocytes and mDC was performed by flow cytometry in a FACSCalibur flow cytometer, after in vitro stimulation with lipopolysaccharide from Escherichia coli plus interferon (IFN)-γ, in the presence of Brefeldina A.

Comparisons between the control and the patient group, and between responders and non-responders to CRT were performed.

**Results:**

Compared to the control group, HF population presented a significantly lower frequency of pDC at baseline and a higher proportion of monocytes and mDC producing IL-6 and IL-1β, both before and 6-months after CRT (T6). There was a remarkable decrease of cMo and an increase of iMo after CRT, only in responders. The responder group also presented higher ncMo values at T6 compared to the non-responder group. Both responders and non-responders presented a decrease in the expression of CD86 in all monocyte and DC populations after CRT. Moreover, in non-responders, the increased frequency of IL-6-producing DC persisted after CRT.

**Conclusion:**

Our study provides new knowledge about the possible contribution of pDC and monocytes subsets to cardiac reverse remodelling and response to CRT. Additionally, CRT is associated with a reduction on CD86 expression by monocytes and DC subsets and in their potential to produce pro-inflammatory cytokines, contributing, at least in part, for the well described anti-inflammatory effects of CRT in HF patients.

**Supplementary Information:**

The online version contains supplementary material available at 10.1186/s12872-023-03574-4.

## Introduction

Chronic heart failure (HF) is a complex and systemic disease [1, 2] characterized by an anomalous structure and function of the heart, resulting in a ventricular filling and/or systolic function impairment [1–4]. Immunological process and inflammation are considered important factors in pathophysiology of heart failure [1, 5, 6], portending a worse functional capacity and a poor prognosis [1, 7].

Cardiac resynchronization therapy (CRT) is a key guideline-recommended treatment for patients with drug-refractory HF, reduced left ventricle ejection fraction (LVEF) and left bundle branch block [8, 9]. Several definitions of CRT response have been used in the literature. Improvement in cardiac function, mainly based on echocardiography demonstration of reverse remodelling, is one of the most widely used definitions. A reduction in left ventricular end-systolic volume (LVESV) greater than or equal to 15% is the most accepted echocardiographic CRT response criterion, given the correlation with clinical outcomes [10]. The beneficial effects of CRT in responders include reverse cardiac remodelling (reduced left ventricular volumes and increased LVEF), improvement of New York Heart Association (NYHA)-based functional status, symptoms and quality of life, reduction of brain natriuretic peptide (BNP), improvement in the six-minute walk test (6MWT), and reduction of mortality and HF hospitalization [11, 12]. In fact, CRT can improve clinical outcomes even in high-risk patients, such as those with type 2 diabetes mellitus [13], when added to the beneficial effects caused by antidiabetic therapies with pleiotropic effects on inflammation and HF [14]. Additionally, the reverse remodelling induced by CRT is related to alterations in the expression of genes and microRNAs (miRs), which regulate cardiac processes involved in cardiac apoptosis, fibrosis, hypertrophy, and angiogenesis, and membrane channelling ionic currents [12, 15].

Previous reports also describe a beneficial effect of CRT on inflammation [16–18], however the relationship between the outcome of CRT-treated patients, cardiac remodelling and immune system response, is not clearly understood. Monocytes and dendritic cells (DC) are pivotal cells in innate and adaptative immune response [19, 20]. While monocytes play a crucial role in host defence, immune regulation, inflammation and tissue repair [5, 19, 21, 22], DC orchestrate T cells response and maintain immune tolerance through different antigens presentation [23, 24]. In fact, there are three different subsets of monocytes – the classical monocytes (cMo) (CD14 +  + /CD16–) which represent about 90% of the total, and two minor CD16 + subsets: the intermediate monocytes (iMo) which express higher levels of CD14 with a lower expression of CD16 (CD14 +  + / CD16 +); and non-classical monocytes (ncMo) that express lower levels of CD14 coupled with high expression of CD16 (CD14 + /CD16 + +) [19, 25, 26], each one with distinct phenotypes and functions [25, 26]. Monocytes seem to be linked to the genesis and development of various cardiovascular events [6, 19, 27]. However, they can also be beneficial, through the production of interleukin (IL)-10, stimulation of angiogenesis and tissue repair [25]. Concerning DC, they can be divided in two major subpopulations according to their haematopoietic origin: myeloid dendritic cells (mDC) and plasmacytoid dendritic cells (pDC) [23, 24, 28]. The role of these antigen presenting cells in HF is not well elucidated [20]. Several human and animal studies in viral and autoimmune myocarditis, myocardial infarction (MI) and dilated cardiomyopathy have described DC as both key inflammatory cells and immunoprotective regulatory cells [20, 24, 28–32].

Given the role of monocytes and DC in tissue repair [33, 34], their contribution in the reverse cardiac remodelling process is conceivable. We hypothesized that monocytes and DC play an important role in cardiac reverse remodelling and CRT response. Therefore, we aimed to assess the potential role of baseline peripheral levels of blood monocytes and DC subsets and their phenotypic and functional activity for CRT response, in HF patients. As a secondary objective, we aimed to evaluate the impact of CRT on peripheral blood monocytes and DC subsets, by quantifying and functionally characterizing cMo, iMo, ncMo, mDC and pDC and comparing the baseline with post CRT results.

## Methods

### Patient population

This is a prospective study enrolling forty-one consecutive patients with advanced HF, undergoing CRT. Implantation of CRT occurred in the tertiary Cardiology Department, Coimbra University Hospital Centre.

Inclusion criteria were defined according to the guideline´s criteria for CRT. Therefore, we restricted patients to those with a class I recommendation for CRT: belonging to class II or III or IV NYHA class; presenting a LVEF ≤ 35%; a QRS ≥ 120 ms with left bundle branch block morphology; and normal sinus rhythm.

The exclusion criteria combined several conditions that could influence or interfere with the inflammatory immune response and bias the results, such as: clinical or biochemical manifestation of concomitant inflammatory disease; regular use of nonsteroidal anti-inflammatory drugs or anticoagulants; active infections; known autoimmune or malignant diseases; severe valvular disease or congenital heart disease; cardiogenic shock; continuously or intermittently intravenous inotropic therapy; pregnancy; deep vein thrombosis or pulmonary embolism; severe peripheral arterial occlusive disease; severe and non-controlled arterial hypertension (systolic blood pressure > 180 mmHg or diastolic > 110 mmHg); comorbidities associated with a life expectancy less than 1 year; recent trauma or surgery (< 1 month); recent major bleeding (< 6 months) requiring blood transfusion; renal insufficiency (creatinine > 2.0 mg/dl); anaemia (haemoglobin < 8.5 g/dl) or thrombocytopenia (< 100000/L); atrial fibrillation; prior arterial coronary bypass surgery; acute coronary syndrome, or percutaneous coronary intervention within three months; previously implanted CRT system; and excessive alcohol consumption or illicit drug abuse [18].

The selection of patients was performed at baseline before the implantation of TRC (T0), with the clinical evaluation and echocardiographic assessment.

At the time of inclusion, all patients were under stable, optimal pharmacological therapy for chronic HF [35, 36].

After six months of follow-up (T6), patients were re-evaluated to assess clinical profile, haematological and chemistry parameters, echocardiographic and inflammatory biomarker changes.

### Echocardiographic evaluation

Standard echocardiography was performed at T0 and T6, using a Vivid 7 (GE Healthcare, Oslo, Norway) and 1.7/3.4-MHz tissue harmonic transducer. Loops and three cardiac cycles were stored digitally and analysed offline using a customized software package (EchoPAC, GE Healthcare). Left ventricular end-diastolic volume (LVEDV), LVESV and LVEF were calculated by the biplane Simpson’s equation in apical four-chamber and two-chamber views [37, 38].

### Definition of response to CRT

The echocardiographic evaluation performed at 6 months follow-up was used to classify the response to CRT. Patients who were still alive and showed at least a 15% reduction in LVESV at 6-month follow-up compared to baseline were considered responders to CRT.

### Healthy control group

The healthy control group (HG) was composed of 11 active healthy volunteers who apparently did not present comorbidities. Inclusion criteria were established considering available and recent analytical results and cardiac exams: history of normal lipid profile and history normal cardiac evaluation. Exclusion criteria were family history of heart disease and/or cardiomyopathy; active infections, inflammatory processes; autoimmune, neoplastic, and allergic diseases; consumption of any drugs or medications that could alter the immune system homeostasis; consumption of alcohol; and inability to understand informed consent.

### Blood samples

Just before implantation of the device, peripheral blood samples were collected in HF patients to determine haematological parameters and for chemistry assessment (including high sensitivity C-reactive protein (hsCRP),BNP and uric acid). In addition, venous samples were taken from patients to analyse inflammatory parameters at T0 and T6.

The same analysis of inflammatory parameters was performed in the HG.

### Quantification and immunophenotypic characterization of circulating dendritic cells and monocyte subsets

Quantification and immunophenotypic characterization of circulating DC and monocytes subsets was assessed using eight-colour combinations of mouse anti-human antibodies: CD11c-allophycocyanin (APC); CD33-peridinin chlorophyll protein (PerCP); CD86-fluorescein isothiocyanate (FITC); CD123-Phycoerythrin (PE); HLA-DR-PE-Cyanine7 (PE-Cy7); CD14 APC-H7; CD16-Pacific Blue (PB) and CD45-Pacific Orange (PO), detailed in Supplementary Table 1.

Briefly, monoclonal antibodies were added to 100 μl of peripheral blood (collected in K3-EDTA) and incubated for 15 min in darkness, at room temperature. Red cell lysis and wash procedures were performed and the remaining cell pellet was resuspended in 0.5 ml of phosphate-buffered saline (PBS) (Gibco, Paisley, Scotland).

#### Flow cytometry data acquisition and analysis

Data acquisition was performed in a FACSCanto™II (BD) flow cytometer equipped with FACSDiva software (version 6.1.2: BD). Samples were acquired with established standardized instrument settings recommended by the Euroflow consortium [39]. Data acquisition was performed in two consecutive steps: in the first step, a total of 1 × 10^5^ events, corresponding to all nucleated cells in the sample, were acquired and results were stored; in the second step, information was stored exclusively for those cells included in a live gate containing HLA-DR^+^ events, with a minimum of 1 × 10^5^ events. Absolute counts were calculated using a dual platform methodology (flow cytometry and haematological cell analyser). Results illustrate the percentage of positive cells within each subset.

**Fig. 1:**
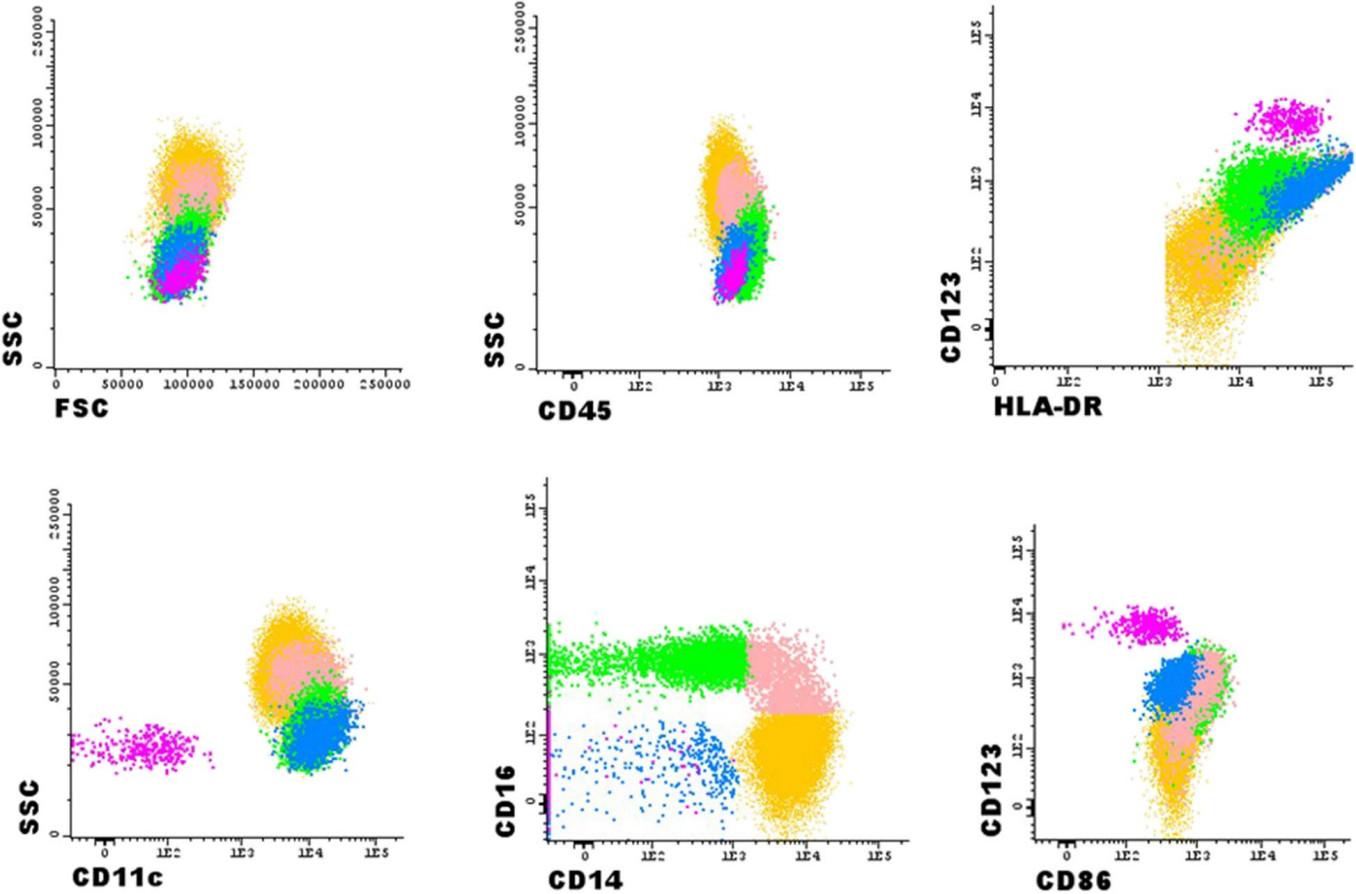
Representative dot plots illustrating the identification of plasmacytoid dendritic cells (pDC) (in pink – based on the bright expression of CD123 and HLA-DR), myeloid dendritic cells (mDC) (in blue – based on the bright expression of CD33 and HLA-DR), classical monocytes (cMo) (in yellow – based on the positive expression of CD14 and negative expression of CD16), intermediate monocyte (iMo) (in light pink—based on the positive expression of both CD14 and CD16), and non-classical monocytes (ncMo) (in green – based on the positive expression of CD16 and dim/negative expression of CD14) in peripheral blood samples, using a combination of eight-colour mouse anti-human antibodies

The DC subsets were identified according to the following phenotypes: mDC as HLA-DR^+bright^CD33^+bright^CD14^−^CD123^+^CD11c^+^ and pDC as HLA-DR^+^CD123^+bright^CD33^−^CD16^−^CD14^−^ [40–42]. The monocytes were identified based on their characteristic FSC/SSC light dispersion properties, strong positivity for CD33, high CD45 expression and CD14 and/or CD16 expression without resorting to the expression of HLA-DR. The cMo were identified as CD33^+++^CD14^+^CD16^−^, iMo as CD14^+^CD16^+^ and ncMo as CD14^±^CD16^+^ [22, 41, 43]. The strategy used for the identification and characterization of DC and monocyte subsets is represented in Fig. 1. The mean fluorescence intensity (MFI) of CD86 was determined in mDC and monocyte subsets and the percentage of CD86^+^ cells, as well as the MFI, in pDC.

### Functional characterization of myeloid dendritic cells and monocytes

In vitro stimulation to evaluate of cytokine production by DC and monocytes was performed as described by Paiva A. et al. [44, 45]. Briefly, a total of 500 μl of each PB sample was diluted l/l (vol/vol), in duplicate, in RPMI-1640 medium (Gibco; Paisley, Scotland, UK), supplemented with 2 mM L-glutamine and incubated at 37 °C in a sterile environment with a 5% CO2 humid atmosphere for 6 h, in the presence of 10 lg/ml of Brefeldin A (Sigma, St. Louis, MO). In addition, 100 ng/ml of lipopolysaccharide (LPS) from Escherichia coli (serotype 055:B5 (Sigma)) plus 100 U/ml of interferon (IFN)-γ; Promega, Madison, WI) were added to one of the tubes (stimulated samples). The other tube only with Brefeldin A was used to evaluate the basal cytokine production by the different subpopulations of monocytes and dendritic cells.

#### Immunofluorescent staining

After incubation period, both stimulated and unstimulated samples were aliquoted in different tubes (200 μl/tube) in order to analyse the expression of each cytokine by monocytes and DC. Dendritic Cell Exclusion Kit-FITC combined with anti-HLA-DR-PerCP and anti-CD33-APC was added to each tube, to identify DC. After gentle mixing, cells were sequentially incubated for 15 min at room temperature, in darkness and washed once in with 2 ml of PBS, (5 min at 540 × g). After discarding the supernatant, cells were fixed and permeabilized with FIX&PERM (Caltag, Hamburg, Germany) according to manufacturer’s instructions and stained with PE-conjugated mAb directed against different human intracytoplasmic cytokines: anti-TNF-α, anti-IL-6 and anti-IL-1β (monoclonal reagents are detailed in Supplementary Table 1). Each anti-cytokine mAb reagent was placed in a separate tube containing either, the stimulated or the unstimulated samples. The tubes were incubated for 15 min at room temperature in darkness. Then, cells were washed and resuspended in 0.5 ml of PBS until they were analysed in a flow cytometer.

#### Flow cytometry data acquisition and analysis

The data acquisition was performed in two consecutive steps in a FACSCalibur flow cytometer (BD, San Jose, USA) equipped with an argon ion laser and a red diode laser. A first acquisition of 2 × 10^4^ events, (corresponding to all nucleated cells present in the sample) was performed, followed by an acquisition on an electronic HLA-DR gate. Data were analysed using the Infinicyt™ software, V.1.5 (Cytognos SL, Salamanca, Spain) and absolute counts were determined using two different instrumentation platforms (flow cytometer and haematological cell analyser).

### Statistical analysis

Statistical analysis was performed using R Core Team (2017). (R: A language and environment for statistical computing. R Foundation for Statistical Computing, Vienna, Austria. URL http://www.R-project.org/), (version 3.4.1). A non-parametric Mann–Whitney U test was used to compare quantitative independent variables. The Wilcoxon signed-rank test was used to compare T0 vs T6 [46]. Results were expressed as median (range). The values used to establish the effect size were 0.20; small, 0.60; moderate, 1.20; large and 2.00; very large [47]. Differences were considered to be statistically significant when *p* value was < 0.05.

To calculate the sample size, the software G*Power 3.1 was used [48]. Prior analysis was performed determining that 35 subjects would be needed for the study (effect size dz:0.7, α error probability:0.05, power:0.80). Additionally, six elements were added to the sample as a matter of convenience.

## Results

### Baseline characteristics

Twenty-eight patients were male and thirteen were female, with ages ranging from 34 to 83 years (mean 61.4 ± 10.4). Regarding chronic medication before CRT, 72.2% of the patients were under angiotensin-converting enzyme (ACE) inhibitors, 19.4% under angiotensin type 1-receptor blockers (ARB), 94.4% under beta-blockers, 66.7% under spironolactone, 97.2% under furosemide, 27.8% under digoxin, 50% under statins, and 13.9% under ivabradine. The rate of diabetic patients was 14.6%.

The HG consisted of eight males and three females with a mean age of 43.4 ± 10.8 years. The average BMI of the HG was 22.8 ± 1.3, and the lipid profile values were 184.3 ± 16.1 mg/dL for cholesterol; 64 ± 8.6 mg/dL for HDL cholesterol; 96.5 ± 11.4 mg/dL for LDL cholesterol and 119.1 ± 24.0 mg/dL for triglycerides.

### Clinical evolution of responders and non-responders to CRT

The clinical characterization of the HF population is detailed in Supplementary Table 2. Most HF patients were in class III according to NYHA classification and mean LVEF was 24.9 ± 6.9%. Through echocardiographic definition, the proportion of responders to CRT was 54%. No patient died or was transplanted in the 6-month follow-up period. After CRT, responders presented significantly lower BNP levels compared to non-responders to CRT.

### Comparison between heart failure patients and healthy group

#### Frequency of monocytes and dendritic cells

The frequency of total monocytes and monocyte subsets expressed by HF patients and the HG is presented in Table 1. Considering the frequency and absolute number of total monocytes, no significant differences were found between HG and HF patients in either moment of evaluation. Regarding monocyte subsets, HF patients presented a significantly lower frequency of cMo at follow-up evaluation, compared to the HG (*P* = 0.006). No other differences were found between the overall HF patient population and the HG in monocytes subsets at either time of evaluation (cMo: 81.00% in the HG versus 79.42% in HF patients (HFP)(T0), *P* = 0.275; iMo: 11.09% in the HG versus 10.81% in HFP(T0), *P* = 0.695 and versus 15.14% in HFP(T6), *P* = 0.065; ncMo: 8.55% in the HG versus 7.52% in HFP(T0), *P* = 0.747 and 11.35% in HFP(T6) *P* = 0.096).

**Table 1:**
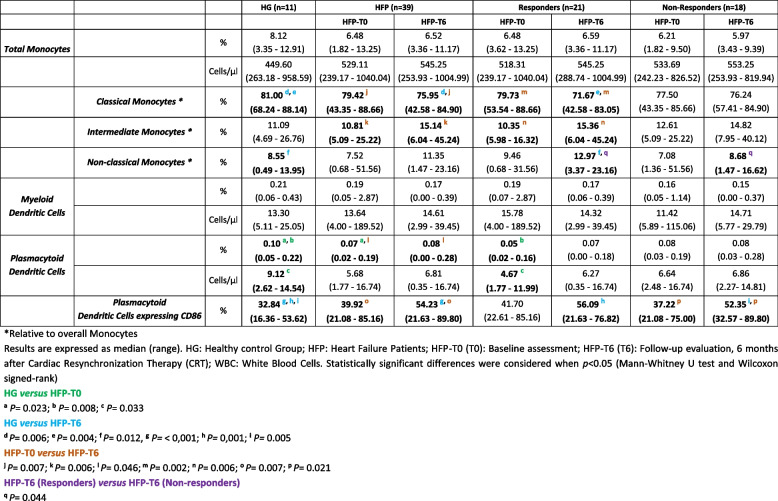
Comparative analysis of the overall monocytes and dendritic cells and their respectively subsets in healthy individuals and patient groups

Regarding total DC and their subsets (Table 1), HF patients presented a significantly lower baseline percentage of pDC compared to the HG (*P* = 0.023). No differences were found in mDC compartment.

#### CD86 expression by monocytes and dendritic cells subpopulations

**Fig. 2:**
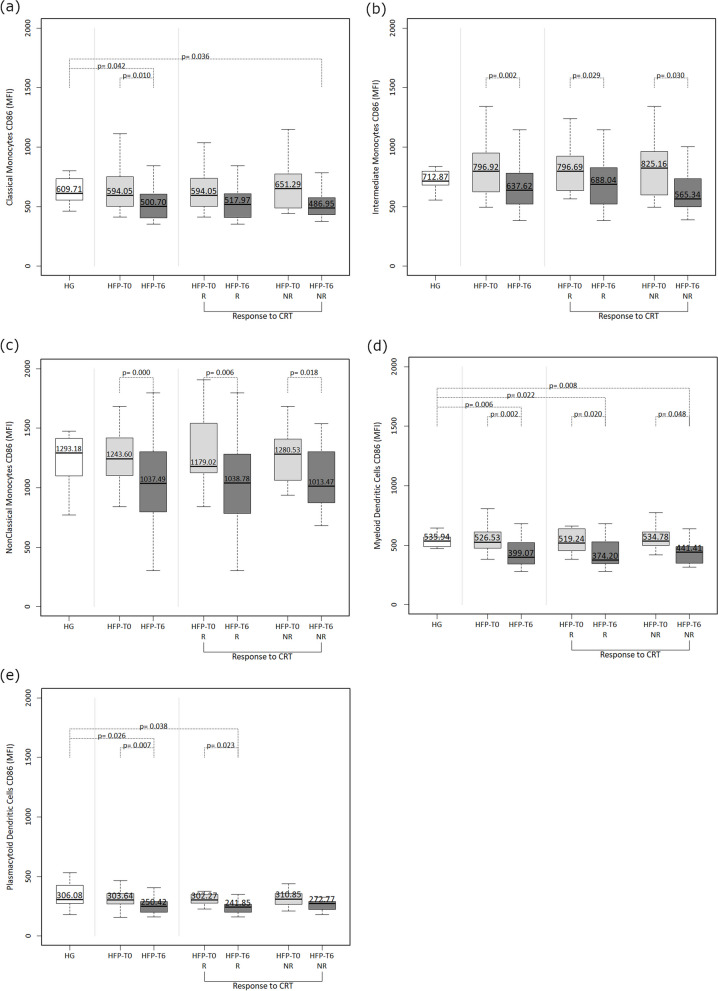
Amount of CD86 *per cell* (based on the mean fluorescence intensity value) expressed by classical monocytes (cMo) (**a**), intermediate monocyte (iMo) (**b**), and non-classical monocytes (ncMo) (**c**), myeloid dendritic cells (mDC) (**d**) and plasmacytoid dendritic cells (pDC) (**e**) in healthy individuals (HG) and heart failure patients (HFP), at baseline assessment (HFP-T0) and 6 months after cardiac resynchronization therapy implantation (HFP-T6). Heart failure patients were distributed: according to response to cardiac resynchronization therapy: responders (R) and non-responders (NR). Statistically significant differences were considered when *p* < 0.05

In both HF and HG, all monocytes subpopulations and mDC expressed the co-stimulatory molecule CD86. Therefore, only the amount of CD86 *per cell* (MFI) was measured (Fig. 2a, b, c and d).

Since not all pDCs express CD86, the frequency of this cell population expressing CD86 is presented in Table 1 and the MFI in Fig. 2e.

At T6, the overall HF population presented a significantly lower MFI of CD86 on cMo (Fig. 2a) and mDC (Fig. 2d), compared with HG.

Considering the pDC subset, HF patients presented a significantly higher percentage of pDC expressing CD86 compared with the HG, at 6-month follow-up (Table 1). However, in the same comparison, the amount of CD86 *per cell* expressed by pDC was lower at follow-up (Fig. 2e) as reported for cMo and mDC.

#### Functional characterization of monocytes and myeloid dendritic cells

**Fig. 3:**
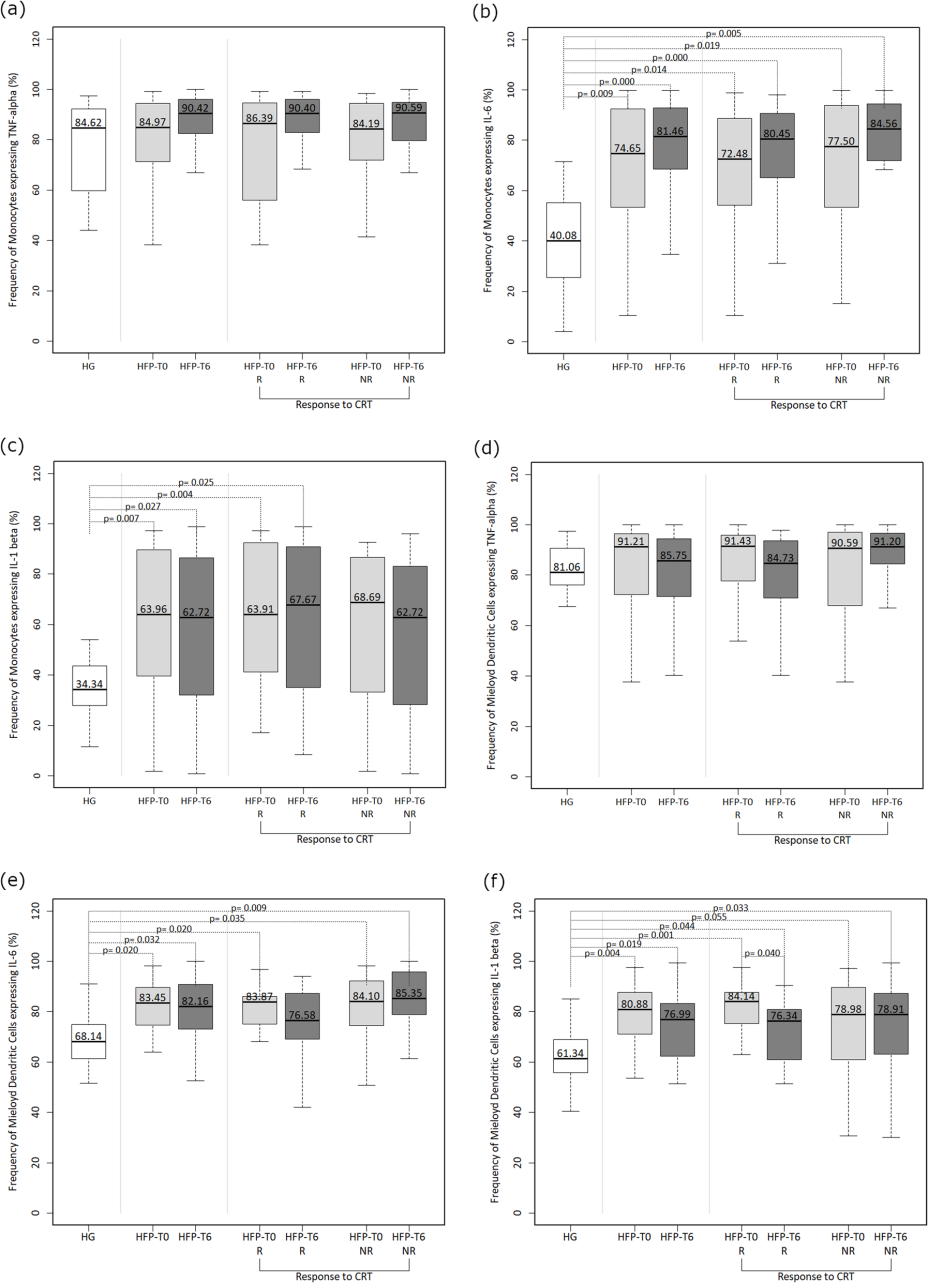
Functional characterization of peripheral monocytes (identified by their characteristic FSC/ SSC light dispersion properties and concomitant expression of CD14, CD33 and HLA-DR) and myeloid dendritic cells (mDC) (Identified by their characteristic FSC/ SSC light dispersion properties, positive expression of CD33 and HLA-DR, and negative expression of all other lineage markers – CD3, CD19, CD56, and CD14 present in the mixture of Dendritic Cell Exclusion Kit). The percentage of monocytes producing TNF-α (**a**), IL-6 (**b**) and IL-1β (**c**) and mDC producing TNF-α (**d**), IL-6 (**e**) and IL-1β (**f**) were evaluated in healthy individuals (HG) and heart failure patients (HFP) at baseline assessment (HFP-T0) and 6 months after cardiac resynchronization therapy implantation (HFP-T6). Heart failure patients were divided according to response to cardiac resynchronization therapy: responders (R) and non-responders (NR). Statistically significant differences were considered when *p* < 0.05

After cell stimulation with LPS and IFN-γ, we only determined the frequency of total monocytes producing the cytokines under study, and not among each monocyte subpopulation. HF patients presented a higher proportion of monocytes and mDC producing IL-6 and IL-1β both before and 6 months after CRT (Fig. 3b, c, e and f), compared with HG. No significant differences were found regarding the frequency of TNF-α-producing monocytes (Fig. 3a) and mDC (Fig. 3d).

### Impact of monocytes and dendritic cells subpopulations on CRT response

#### Frequency of monocytes and dendritic cells

When we divide patients according to CRT response, only responders showed significantly lower levels of cMo after CRT than HG (*P* = 0.004, Table 1). Moreover, responders to CRT, but not non-responders, presented a significant reduction of cMo levels from baseline to post-CRT evaluation. The responder group also showed a significantly higher percentage of ncMo at follow-up compared to the HG (*P* = 0.012, Table 1).

On the other hand, after CRT, non-responders maintained similar levels of cMo and ncMo by comparison with the HG (cMo: HG = 81.00% versus non-responders (T6) = 76.24%, *P* = 0.056 and ncMo: HG = 8.55% versus non-responders (T6) = 8.68%, *P* = 0.902). Concerning the frequency of iMo, no differences were observed in this comparison with HG.

In relation to DC subpopulations, the significantly lower percentage of pDC compared to the HG was only observed in the responder group (pDC: HG = 0.10% versus responders(T0) = 0.05%, *P* = 0.008; and absolute values pDC: HG = 9.12cell/µl versus responders(T0) = 4.67 cell/µl*, P* = 0.033, Table 1). No differences were found in the mDC subset.

In the analysis between the two moments of evaluation (T0 and T6), the overall HF population showed a significantly decrease in the percentage of cMo at follow-up, and a significant increase in the frequency of iMo compared to baseline assessment (Table 1). Of note is that these significant differences were only observed in responders to CRT (Table 1); (cMo(non-responders): T0 = 77.50% versus T6 = 76.24%, *P* = 0.632; iMo(non-responders): T0 = 12.61% versus T6 = 14.82%, *P* = 0.298).

Regarding the ncMo subset, no differences were observed in the overall HF population between T0 and T6. However, responders to CRT presented higher levels of ncMo than non-responders at 6-month follow-up (*P* = 0.044, Table 1). Here, it was observed that, at 6-month follow-up, responders presented not only higher values of ncMo to the detriment of cMo, but also increased iMo frequency.

Considering DC, we only found one significant difference between baseline and follow-up evaluation: HF patients exhibited a higher frequency of pDC at follow-up (*P* = 0.046, Table 1).

#### Expression of CD86 by monocytes and dendritic cells

As shown in Table 1, there was remarkably higher frequency of CD86-expressing pDC after CRT in comparison with the HG, in both responders (*P* = 0.001) and non-responders (*P* = 0.005). However, as described previously, the MFI of CD86 decreased after CRT in HF patients compared with the HG, but only reaching significance in responders (Fig. 2e) (pDC CD86 MFI: HG = 306.08 versus non-responders(T6) = 272.77, *P* = 0.069).

Compared with HG, the amount of CD86 *per cell* in cMo was significantly lower after CRT especially in non-responders (Fig. 2a) (cMo CD86 MFI: HG = 609.71 *versus* responders(T6) = 517.97, *P* = 0.113). In the mDC subset this decrease was seen in both response groups (Fig. 2d).

Comparing the baseline with follow-up (T0 versus T6) there was a significantly lower amount of CD86 *per cell* expressed in all monocytes and DC subpopulations after CRT. This pattern of decreased MFI of CD86 was seen independently of CRT response.

Considering the frequency of pDC expressing CD86, at 6-month follow-up the overall HF population presented a higher percentage of these cells compared to baseline assessment. However, this difference was mostly observed in non-responders (*P* = 0.021, Table 1).

#### Functional characterization of monocytes and dendritic cells

No significant differences were found between HF patients and the HG, nor between responders and non-responders to CRT or between baseline and follow-up (T0 versus T6) regarding the frequency of TNF-α-producing monocytes (Fig. 3a).

In the initial evaluation and even after CRT, both responders and non-responders presented a higher proportion of IL-6-producing monocytes, compared with the HG (Fig. 3b). Likewise, responders also showed significantly more IL-1β-producing monocytes, at both times of evaluation, relative to the HG (Fig. 3c).

No significant differences were found regarding the frequency of IL6 and IL1β-producing monocytes, when comparing baseline with post-CRT (T0 versus T6) nor when comparing responders and non-responders (R versus NR) (Fig. 3b, 3c).

Regarding TNF-α-producing DC, we also found no significant differences between responder or non-responder patients and the HG (Fig. 3d). However, both patient groups presented higher baseline levels of DC expressing IL-6 compared to the HG. Nonetheless, after CRT, this difference only persisted in the non-responder group (Fig. 3e).

In addition, the proportion of IL-1β-producing DC was significantly increased in patients compared to the HG at both T0 and T6 evaluation times. Responders and non-responders presented a higher proportion of these cells compared to the HG. Curiously, the responder group showed a lower percentage of IL-1β producing DC at follow-up compared to the baseline (Fig. 3f).

## Discussion

Monocytes and DC have been implicated in the pathogenesis of HF as well as in its prognosis [19, 21, 28]. To the best of our knowledge, this is the first study to assess the potential role of DC subpopulations and monocyte subsets on cardiac reverse remodelling and CRT response.

In the first comparison between the HG and the HF population, the main findings are the lower frequency of pDC in HF patients before CRT and the lower frequency of cMo at the 6-month follow-up. Previous studies on whole blood DC and monocyte counts in HF patients have shown dissimilar results [6, 23, 25, 28, 29, 49, 50]. Some studies describe an increase in mDC subpopulation with unchanged levels of pDC [49] or comparable levels of both DC between HF patients and controls [23]. Nonetheless, a recent study performed by Pistulli R et al. (2016), reports lower counts of circulating mDC in patients with advanced HF [29]. Furthermore, Barisione C. et al. describe an increase in iMo and a decrease in ncMo subsets in HF patients compared to the control group [6] and in the latest study performed by Ptaszynska-Kopczynska K. et al. (2021) a decrease in circulating ncMo was only found in patients with advanced HF [25]. These distinct results may be due to the heterogeneity of the patient populations in these previous studies, linked to different inclusion and exclusion criteria, HF severity, HF aetiology and sample size. Of note is that our study has the advantage of enrolling a homogeneous population with advanced HF and class I indication for CRT.

Regarding the expression of CD86, HF patients showed similar levels of the amount of CD86 *per cell* at baseline compared to the HG, which suggests that the expression of this co-stimulatory molecule by the antigen-presenting cells under study is unaffected in HF. However, after CRT, patients presented a lower amount of CD86 *per cell* on cMo and mDC, which indicates an immunomodulation of the expression of this important co-stimulatory molecule. At the same time, although the frequency of pDC expressing CD86 increases after CRT (which could indicate that these cells may be peripherally activated, with a likely increased capacity to produce robust amounts of type I IFNs), the MFI of CD86 on pDC was also lower in patients than in healthy subjects. At this point, it appears that CRT can exert an impact on the amount of CD86 expressed by monocytes and DC.

As expected, at baseline, HF patients showed a higher frequency of Mo and mDC producing IL-6 and IL-1β compared to the HG. These higher levels of pro-inflammatory cytokine-producing cells reflect the inflammatory state of HF. This difference persisted after CRT, suggesting that biventricular pacing is not able to decrease the frequency of these cells to normal values. Considering that the various monocyte subsets produce TNF-α, IL-6, IL-1β and that these inflammatory cytokines are important markers of active disease and HF prognosis [51], our study shows that CRT does not modulate the pro-inflammatory capacity of these cells, which may compromise the long-term response to CRT and even the overall survival of patients.

Regarding the comparison between responders and non-responders to CRT, the fact that responders showed significantly lower levels of cMo and higher levels of ncMo after CRT than the HG but that non-responders did not, suggests that the reduction of circulating cMo and the increase in ncMo play a role in CRT response. Some studies have shown that the inflammatory response induced by the innate immune system can be physiological and results in the upregulation of cytoprotective responses that allow the heart to adapt to stress [52, 53]. Therefore, it is tempting to speculate that the cMo reduction observed after CRT, exclusively in responders, indicates an increased recruitment of circulating monocytes to the injury site and a beneficial effect on reverse remodelling, or, on the other hand, it may be due to an increase in the ncMo subset, which after exerting their local function, emerge from the cardiac tissue into the peripheral blood circulation to die in the spleen [54].

Another important finding of the present study is the remarkable decrease in cMo and increase in iMo, from baseline to post-CRT, which was only due to responders. Furthermore, the responder group also presented higher ncMo values at follow-up compared to the non-responder group. Taken together, the increase in the frequency of ncMo and iMo and decrease in cMo in responders suggests a participation of these cells in cardiac reverse remodelling and CRT response. Our results are consistent with those of Ptaszynska-Kopczynska K. et al. (2021) [25], who describe an increase in ncMo and iMo subsets with a consequent reduction of cMo in HF patients after CRT. However, in that study the subdivision of patients according to CRT response was not carried out. Our study included an additional analysis based on responders and non-responders that distinguishes it from prior studies. Functionally iMo are involved in the induction of natural repair mechanisms such as regulation of immune response, pro-angiogenesis, and tissue regeneration [25, 26, 55, 56], while ncMo are known for their patrolling behaviour, surveying the endothelium for signs of inflammation or damage [26, 57]. In addition, despite being associated with inflammatory disease progression, ncMo are crucial for vascular homeostasis, removing damaged cells and debris from the endothelium, displaying an important role in wound-healing, collagen deposition, angiogenesis, and resolution of inflammation by linking innate to adaptive immune response [57–60]. Moreover, a recent study by van de Bossche et al. [54] describes that, after completing their tissue-cleaning task, monocytes can migrate through the lymphatic system into the bloodstream, allowing phagolysosomal content to be evaluated and damage detection in this tissue to be used as a marker for therapeutic monitoring of several disease conditions. In this context, the differences in the frequency of peripheral ncMo observed between responders and non-responders after CRT can be explained by this behaviour of ncMo in the process of healing and cleaning of cardiac tissue and migration to peripheral blood.

Concerning cytokine production by monocytes, even responders showed an increased frequency of these cells expressing IL-6 and IL-1β at both times of evaluation, suggesting once again that CRT was not able to interfere with the functional inflammatory ability of monocytes. Interestingly, in the analysis of IL-6-producing mDC, although both responders and non-responders showed a higher frequency of cells at baseline, after CRT this difference disappears in responders. In this context, our results suggest that, after reverse remodelling, mDC might display lower functional inflammatory capacity. Furthermore, although CRT fails to reduce the inflammatory capacity of monocytes, the responder group showed a lower frequency of IL1β-producing mDC at follow-up compared to baseline, as well as lower values of IL-6 producing-mDC compared to non-responders. At this point, it can be concluded that there is also a tendency for CRT to suppress the inflammation produced by DC, but inflammatory values after stimulation remain higher than the HG.

Regarding the DC subpopulations, only responders presented lower frequency and absolute values of pDC at baseline compared to the HG. As producers of massive amounts of type-I IFNs when activated, pDC have been implicated in the development of autoimmune and inflammatory diseases [61–63]. In our work, the low frequency of pDC at baseline seems to be an indicator of positive response to CRT.

Another remarkable result of our study is the decrease in CD86 expression by monocytes and DC after CRT compared to the initial evaluation, suggesting an immune modulating role of CRT, whether in responder or non-responder patients, due to a lower ability of monocytes and DC to provide the second antigen-independent co-signal to T cells, which may compromise the adaptive immune response on the inflammatory process in HF. Interestingly, the increased frequency of pDC expressing CD86 observed in the general HF population at 6-month follow-up was primarily seen in non-responders.

Our group recently published a study on Treg cells in patients with HF. We showed that peripheral blood Treg cells were decreased in patients and remained reduced after CRT [64]. On the other hand, MI studies performed in animal models describe the recruitment of these tolerogenic cells to the heart in order to suppress the inflammatory response [65, 66]. Furthermore, it is described that Treg cells can not only improve healing after MI but also trigger monocyte differentiation [66]. In this sense, after CRT, the migration of Treg cells to the failing heart may continue to occur, which could, at least in part, explain the decrease in CD86 expression by monocytes and DC and the differentiation of monocytes into iMo and ncMo.

The present study has some limitations. Despite being a homogeneous population (with advanced HF submitted to CRT), one important limitation of our study is the small sample size, especially in comparisons between subgroups. Other studies with larger samples are needed to prove whether the reduction of monocytes and DC is in fact related to the positive response to CRT. Another limitation is the short follow-up period. We did not experience whether the possible anti-inflammatory effect of CRT is sustained over time.

In conclusion, our research suggests that the innate immune system participates in cardiac reverse remodelling and response to CRT. HF patients with less pDC appear to be more prone to respond to CRT, and the decrease in cMo values (which have proinflammatory effects) with the increase in iMo (with beneficial and anti-inflammatory properties) and ncMo (important in wound-healing) after CRT seem to be related to successful reverse cardiac remodelling. Furthermore, CRT is associated with a reduction in the amount of CD86 expressed by monocytes and DC subsets and in their potential to produce pro-inflammatory cytokines, which may influence the connection between the innate and adaptive immune response, contributing, at least in part, to the previously described anti-inflammatory effects of CRT.

### Supplementary Information

**Additional file 1: Supplementary Table 1.** Monoclonal antibody reagents used for the immunophenotypic and functional characterization of monocytes and dendritic cells.

**Additional file 2: Supplementary Table 2.** Clinical characterization of responders and non-responders to CRT.

## Data Availability

The main data were obtained at FACSCalibur and FACSCanto™II flow cytometer and were presented within the article. For statistical analysis, and graphical construction,*.xlsx*,*.txt* and*.r* formats were used. The data underlying this article cannot be shared publicly due to the presence of personal identification of research participants, which could compromise privacy rights. The data supporting the results will be shared on reasonable request to the corresponding author, Paiva A.

## References

[1] Reina-Couto M, Pereira-Terra P, Quelhas-Santos J, Silva-Pereira C, Albino-Teixeira A, Sousa T (2021). Inflammation in human heart failure: major mediators and therapeutic targets. Front Physiol.

[2] Zhang Y, Bauersachs J, Langer HF (2017). Immune mechanisms in heart failure. Eur J Heart Fail.

[3] Na-A UK, Atherton JJ, Bauersachs J (2016). ESC Guidelines for the diagnosis and treatment of acute and chronic heart failure. Eur Heart J.

[4] Mari D, Di Berardino F, Cugno M (2002). Chronic heart failure and the immune system. Clin Rev Allergy Immunol.

[5] Wrigley BJ, Lip GY, Shantsila E (2011). The role of monocytes and inflammation in the pathophysiology of heart failure. Eur J Heart Fail.

[6] Barisione C, Garibaldi S, Ghigliotti G (2010). CD14CD16 monocyte subset levels in heart failure patients. Dis Markers.

[7] Fu M (2009). Inflammation in chronic heart failure: what is familiar, what is unfamiliar?. Eur J Heart Fail.

[8] McDonagh TA, Metra M, Adamo M (2021). 2021 ESC Guidelines for the diagnosis and treatment of acute and chronic heart failure: Developed by the Task Force for the diagnosis and treatment of acute and chronic heart failure of the European Society of Cardiology (ESC) With the special contribution of the Heart Failure Association (HFA) of the ESC. Eur Heart J.

[9] Glikson M, Nielsen JC, Kronborg MB (2022). 2021 ESC Guidelines on cardiac pacing and cardiac resynchronization therapy: Developed by the Task Force on cardiac pacing and cardiac resynchronization therapy of the European Society of Cardiology (ESC) With the special contribution of the European Heart Rhythm Association (EHRA). EP Europace.

[10] Nakai T, Ikeya Y, Kogawa R (2021). What are the expectations for cardiac resynchronization therapy? A validation of two response definitions. J Clin Med.

[11] Ocaranza MP, Jalil JE, Altamirano R (2021). Reverse remodeling in human heart failure after cardiac resynchronization therapy is associated with reduced RHO-kinase activation. Front Pharmacol.

[12] Sardu C, Barbieri M, Rizzo MR, Paolisso P, Paolisso G, Marfella R (2016). Cardiac resynchronization therapy outcomes in type 2 diabetic patients: role of microRNA changes. J Diabetes Res.

[13] Sardu C, Barbieri M, Santamaria M (2017). Multipolar pacing by cardiac resynchronization therapy with a defibrillators treatment in type 2 diabetes mellitus failing heart patients: impact on responders rate, and clinical outcomes. Cardiovasc Diabetol.

[14] Sardu C, Paolisso P, Sacra C (2018). Cardiac resynchronization therapy with a defibrillator (CRTd) in failing heart patients with type 2 diabetes mellitus and treated by glucagon-like peptide 1 receptor agonists (GLP-1 RA) therapy vs. conventional hypoglycemic drugs: arrhythmic burden, hospitalizations for heart failure, and CRTd responders rate. Cardiovasc Diabetol.

[15] Sardu C, Massetti M, Scisciola L (2022). Angiotensin receptor/Neprilysin inhibitor effects in CRTd non-responders: from epigenetic to clinical beside. Pharmacol Res.

[16] Osmancik P, Herman D, Stros P, Linkova H, Vondrak K, Paskova E (2013). Changes and prognostic impact of apoptotic and inflammatory cytokines in patients treated with cardiac resynchronization therapy. Cardiology.

[17] Ptaszynska-Kopczynska K, Sawicka E, Marcinkiewicz-Siemion M (2020). Chemokines profile in patients with chronic heart failure treated with cardiac resynchronization therapy. Adv Med Sci.

[18] Martins S, Carvalheiro T, Laranjeira P (2019). Impact of cardiac resynchronization therapy on circulating IL-17 producing cells in patients with advanced heart failure. J Interv Card Electrophysiol.

[19] Elchinova E, Teubel I, Roura S (2018). Circulating monocyte subsets and heart failure prognosis. PLoS One.

[20] Pistulli R, Andreas E, König S (2020). Characterization of dendritic cells in human and experimental myocarditis. ESC Heart Failure.

[21] Glezeva N, Horgan S, Baugh JA (2015). Monocyte and macrophage subsets along the continuum to heart failure: misguided heroes or targetable villains?. J Mol Cell Cardiol.

[22] Ziegler-Heitbrock L, Ancuta P, Crowe S (2010). Nomenclature of monocytes and dendritic cells in blood. Blood J Am Soc Hematol.

[23] Athanassopoulos P, Balk AH, Vaessen LM (2009). Blood dendritic cell levels and phenotypic characteristics in relation to etiology of end-stage heart failure: implications for dilated cardiomyopathy. Int J Cardiol.

[24] Pistulli R, König S, Drobnik S (2013). Decrease in dendritic cells in endomyocardial biopsies of human dilated cardiomyopathy. Eur J Heart Fail.

[25] Ptaszyńska-Kopczyńska K, Eljaszewicz A, Marcinkiewicz-Siemion M (2021). Monocyte subsets in patients with chronic heart failure treated with cardiac resynchronization therapy. Cells.

[26] Wong KL, Yeap WH, Tai JJY, Ong SM, Dang TM, Wong SC (2012). The three human monocyte subsets: implications for health and disease. Immunol Res.

[27] Idzkowska E, Eljaszewicz A, Miklasz P, Musial WJ, Tycinska AM, Moniuszko M (2015). The role of different monocyte subsets in the pathogenesis of atherosclerosis and acute coronary syndromes. Scand J Immunol.

[28] Dieterlen M-T, John K, Reichenspurner H, Mohr FW, Barten MJ (2016). Dendritic cells and their role in cardiovascular diseases: a view on human studies. J Immunol Res.

[29] Pistulli R, Hammer N, Rohm I (2016). Decrease of circulating myeloid dendritic cells in patients with chronic heart failure. Acta Cardiol.

[30] Maekawa Y, Mizue N, Chan A (2009). Survival and cardiac remodeling after myocardial infarction are critically dependent on the host innate immune interleukin-1 receptor-associated kinase-4 signaling: a regulator of bone marrow-derived dendritic cells. Circulation.

[31] Anzai A, Anzai T, Nagai S (2012). Regulatory role of dendritic cells in postinfarction healing and left ventricular remodeling. Circulation.

[32] Cai G, Wang H, Qin Q (2009). Amelioration of myocarditis by HVEM-overexpressing dendritic cells through induction of IL-10-producing cells. Cardiovasc Res.

[33] Wynn TA, Vannella KM (2016). Macrophages in tissue repair, regeneration, and fibrosis. Immunity.

[34] Julier Z, Park AJ, Briquez PS, Martino MM (2017). Promoting tissue regeneration by modulating the immune system. Acta Biomater.

[35] Ramani GV, Uber PA, Mehra MR (2010). Chronic heart failure: contemporary diagnosis and management. Mayo Clin Proc..

[36] Jameel MN, Zhang J (2009). Heart failure management: the present and the future. Antioxid Redox Signal.

[37] Cristóvão G, Milner J, Sousa P (2020). Improvement in circulating endothelial progenitor cells pool after cardiac resynchronization therapy: increasing the list of benefits. Stem Cell Res Ther.

[38] Kydd AC, Khan FZ, Ring L, Pugh PJ, Virdee MS, Dutka DP (2014). Development of a multiparametric score to predict left ventricular remodelling and prognosis after cardiac resynchronization therapy. Eur J Heart Fail.

[39] Kalina T, Flores-Montero J, Van Der Velden V (2012). EuroFlow standardization of flow cytometer instrument settings and immunophenotyping protocols. Leukemia.

[40] Collin M, McGovern N, Haniffa M (2013). Human dendritic cell subsets. Immunology.

[41] Monteiro A, Rosado P, Rosado L, Fonseca AM, Coucelo M, Paiva A (2021). Alterations in peripheral blood monocyte and dendritic cell subset homeostasis in relapsing-remitting multiple sclerosis patients. J Neuroimmunol.

[42] Zhang Y, Zhang C (2010). Role of dendritic cells in cardiovascular diseases. World J Cardiol.

[43] Skrzeczyńska-Moncznik J, Bzowska M, Loseke S, Grage-Griebenow E, Zembala M, Pryjma J (2008). Peripheral blood CD14high CD16+ monocytes are main producers of IL-10. Scand J Immunol.

[44] Carvalheiro T, Velada I, Valado A (2012). Phenotypic and functional alterations on inflammatory peripheral blood cells after acute myocardial infarction. J Cardiovasc Transl Res.

[45] Henriques A, Inês L, Carvalheiro T (2012). Functional characterization of peripheral blood dendritic cells and monocytes in systemic lupus erythematosus. Rheumatol Int.

[46] Vetter TR, Mascha EJ (2018). Unadjusted bivariate two-group comparisons: when simpler is better. Anesth Analg.

[47] Hopkins W, Marshall S, Batterham A, Hanin J (2009). Progressive statistics for studies in sports medicine and exercise science. Med Sci Sports Exerc.

[48] Kang H (2021). Sample size determination and power analysis using the G* Power software. J Educ Eval Health Prof.

[49] Athanassopoulos P, Vaessen LM, Maat AP, Balk AH, Weimar W, Bogers AJ (2004). Peripheral blood dendritic cells in human end-stage heart failure and the early post-transplant period: evidence for systemic Th1 immune responses. Eur J Cardiothorac Surg.

[50] Sugi Y, Yasukawa H, Kai H (2011). Reduction and activation of circulating dendritic cells in patients with decompensated heart failure. Int J Cardiol.

[51] Shahid F, Lip GY, Shantsila E (2018). Role of monocytes in heart failure and atrial fibrillation. J Am Heart Assoc.

[52] Besse S, Nadaud S, Balse E, Pavoine C (2022). Early protective role of inflammation in cardiac remodeling and heart failure: focus on TNFα and resident macrophages. Cells.

[53] Mann DL (2003). Stress-activated cytokines and the heart: from adaptation to maladaptation. Annu Rev Physiol.

[54] van den Bossche WB, Vincent AJ, Teodosio C (2021). Monocytes carrying GFAP detect glioma, brain metastasis and ischaemic stroke, and predict glioblastoma survival. Brain communications.

[55] Eljaszewicz A, Kleina K, Grubczak K (2018). Elevated numbers of circulating very small embryonic-like stem cells (VSELs) and intermediate CD14++ CD16+ monocytes in IgA nephropathy. Stem cell reviews and reports.

[56] Eljaszewicz A, Bolkun L, Grubczak K (2018). Very small embryonic-like stem cells, endothelial progenitor cells, and different monocyte subsets are effectively mobilized in acute lymphoblastic leukemia patients after G-CSF treatment. Stem Cells Int.

[57] Charach G, Rogowski O, Karniel E, Charach L, Grosskopf I, Novikov I (2019). Monocytes may be favorable biomarker and predictor of long-term outcome in patients with chronic heart failure: a cohort study. Medicine.

[58] Tahir S, Steffens S (2021). Nonclassical monocytes in cardiovascular physiology and disease. Am J Physiol Cell Physiol.

[59] Thomas G, Tacke R, Hedrick CC, Hanna RN (2015). Nonclassical patrolling monocyte function in the vasculature. Arterioscler Thromb Vasc Biol.

[60] Kologrivova I, Suslova T, Koshelskaya O, Trubacheva O, Haritonova O, Vinnitskaya I (2020). Frequency of monocyte subsets is linked to the severity of atherosclerosis in patients with ischemic heart disease: a case-control study. Biomedicine.

[61] Chistiakov DA, Orekhov AN, Sobenin IA, Bobryshev YV (2014). Plasmacytoid dendritic cells: development, functions, and role in atherosclerotic inflammation. Front Physiol.

[62] Ye Y, Gaugler B, Mohty M, Malard F (2020). Plasmacytoid dendritic cell biology and its role in immune-mediated diseases. Clin Transl Immunol.

[63] Guillerey C, Mouriès J, Polo G (2012). Pivotal role of plasmacytoid dendritic cells in inflammation and NK-cell responses after TLR9 triggering in mice. Blood J Am Soc Hematol.

[64] Martins S, António N, Carvalheiro T (2023). Reduced numbers of regulatory T cells in chronic heart failure seems not to be restored by cardiac resynchronization therapy. BMC Cardiovasc Disord.

[65] Saxena A, Dobaczewski M, Rai V (2014). Regulatory T cells are recruited in the infarcted mouse myocardium and may modulate fibroblast phenotype and function. Am J Physiol Heart Circ Physiol.

[66] Weirather J, Hofmann UD, Beyersdorf N (2014). Foxp3+ CD4+ T cells improve healing after myocardial infarction by modulating monocyte/macrophage differentiation. Circ Res.

