# Supplementary material for: Role of monocytes and dendritic cells in cardiac reverse remodelling after cardiac resynchronization therapy
Source: BMC Cardiovasc Disord. 2023 Nov 15;23:558. doi: 10.1186/s12872-023-03574-4 (PMC10652525; doi:10.1186/s12872-023-03574-4)
Supplement: Supplementary file 1 — Additional file 1: Supplementary Table 1. Monoclonal antibody reagents used for the immunophenotypic and functional characterization of monocytes and dendritic cells. [file 12872_2023_3574_MOESM1_ESM.docx]

**Supplementary Table 1** – Monoclonal antibody reagents used for the immunophenotypic and functional characterization of monocytes and dendritic cells.

| **Monoclonal antibodies** | **Fluorochromes** | **Clone** | **Commercial Source** |
| --- | --- | --- | --- |
| **CD11c** | APC | SHCL-3 | BDB |
| **CD33** | PerCP | P67.6 | BDB |
| **CD86** | FITC | 2331 (FUN-1) | BD Pharmingen |
| **CD123** | PE | 9F5 | BDB |
| **HLA-DR** | PE-Cy7 | L243 | BDB |
| **CD14** | APC-H7 | MφP9 | BDB |
| **CD16** | PB | 3G8 | BD Pharmingen |
| **CD45** | PO | HI30 | Invitrogen |
| **Dendritic Cell Exclusion Kit**  (Mixture of anti-CD3,  CD56, CD19, and CD14) | FITC |  | Cytogonos, Salamanca, Spain |
| **HLA-DR** | PerCP | L243 | BDB, San Jose, USA |
| **CD33** | APC | clone P67.6 | BDB, San Jose, USA |
| **TNF-α** | PE | Mab11 | Pharmingen, San Diego, USA |
| **IL-6** | PE | clone MQ2- 6A | Pharmingen, San Diego, USA |
| **IL-1β** | PE | clone AS10 | BDB, San Jose, USA |
