# Supplementary material for: Role of monocytes and dendritic cells in cardiac reverse remodelling after cardiac resynchronization therapy
Source: BMC Cardiovasc Disord. 2023 Nov 15;23:558. doi: 10.1186/s12872-023-03574-4 (PMC10652525; doi:10.1186/s12872-023-03574-4)
Supplement: Supplementary file 2 — Additional file 2: Supplementary Table 2. Clinical characterization of responders and non-responders to CRT. [file 12872_2023_3574_MOESM2_ESM.docx]

**Supplementary Table 2:** Clinical characterization of responders and non-responders to CRT

|  | Global Population  Mean ± SD  (n=41) | Responders  Mean ± SD  (n=22) | Non-Responders  Mean ± SD  (n=19) | *P Value*  *Responders*  *vs*  *Non-responders* |
| --- | --- | --- | --- | --- |
| **Baseline assessment** | | | | |
| Gender (Male/Female) | 28 / 13 | 15 / 7 | 13 / 6 | 1 |
| Aetiology (Non-Ischemic/Ischemic) | 30 / 11 | 18 / 4 | 12 / 7 | 0,418 |
| NYHA (II/III/IV) | 8/ 29/ 4 | 4/ 17/ 1 | 4/ 12 /3 | 0,465 |
| Age (years) | 61.4 ± 10.5 | **65.2 ± 9.6** | **56.9 ± 9.8** | **0.015** |
| LVEF (%) | 24.9 ± 6.9 | 23.8 ± 6.5 | 26.3 ± 7.4 | 0.309 |
| LVESV (mL) | 190.2 ± 84.9 | 180.1 ± 55.3 | 202.1 ± 111.0 | 0.903 |
| LVEDV (mL) | 244.2 ± 83.9 | 233.3 ± 58.9 | 257.1 ± 106.8 | 0.583 |
| QRS | 148.4 ± 30.6 | 144.5 ± 22.1 | 151.4 ± 36.6 | 0.866 |
| Total Leukocytes (x10^3^ µl) | 8.4 ± 1.7 | 8.1 ± 1.6 | 8.7 ± 1.8 | 0.242 |
| hsCRP (mg/L) | 5.6 ± 6.0 | 5.2 ± 5.1 | 5.9 ± 6.9 | 0.934 |
| BNP (pg/mL) | 362.8 ± 358.7 | 264.3 ± 214.8 | 461.3 ± 448.1 | 0.362 |
| Glucose (mg/dL) | 109.9 ± 41.9 | 97 ± 31.9 | 100 ± 50.8 | 0.286 |
| Uric Acid (mg/dL) | 6.0 ± 1.7 | 5.6 ± 1.5 | 6.5 ± 1.9 | 0.231 |
| **After CRT** | | | | |
| Total Leukocytes (x10^3^ µl) | 8.3 ± 1.8 | 8.1 ± 1.5 | 8.6 ± 2.1 | 0.220 |
| LVEF (%) | 33.9 ± 10.8 | **39.1 ± 9.8** | **27.6 ± 8.4** | **0.001** |
| LVESV (mL) | 151.4 ± 96.0 | **100.4 ± 36.7** | **215.1 ± 109.6** | **<0.001** |
| LVEDV (mL) | 220.2 ± 108.5 | **168.9 ± 50.3** | **284.4 ± 127.9** | **0.001** |
| hsCRP (mg/L) | 4.1 ± 4.6 | 2.6 ± 1.8 | 6.2 ± 6.4 | 0.288 |
| BNP (pg/mL) | 245.3 ± 334.6 | **139.6 ± 164.1** | **403.9 ± 456.0** | **0.043** |
| SD: standard deviation; NYHA: New York Heart Association; LVEF: Left Ventricular Ejection Fraction; LVESV: Left Ventricular End-Systolic Volume; LVEDV: Left Ventricular End-Diastolic Volume; hsCRP: High Sensitivity C-Reactive Protein; BNP: B-type natriuretic peptide. | | | | |
